# First Evidence of Co-Circulation of Emerging *Leishmania martiniquensis*, *Leishmania orientalis*, and *Crithidia* sp. in *Culicoides* Biting Midges (Diptera: Ceratopogonidae), the Putative Vectors for Autochthonous Transmission in Southern Thailand

**DOI:** 10.3390/tropicalmed7110379

**Published:** 2022-11-15

**Authors:** Nopporn Songumpai, Chulaluk Promrangsee, Preudtipong Noopetch, Padet Siriyasatien, Kanok Preativatanyou

**Affiliations:** 1 Division of Infectious Diseases, Department of Internal Medicine, Hatyai Hospital, Songkhla 90110, Thailand; zea_me@hotmail.com (N.S.); jasommhai@gmail.com (P.N.); 2 Interdisciplinary Program of Biomedical Sciences, Graduate School, Chulalongkorn University, Bangkok 10330, Thailand; famezaflamingoclub@hotmail.com; 3 Center of Excellence in Vector Biology and Vector-Borne Disease, Department of Parasitology, Faculty of Medicine, Chulalongkorn University, Bangkok 10330, Thailand; padet.s@chula.ac.th; 4 Department of Parasitology, Faculty of Medicine, Chulalongkorn University, Bangkok 10330, Thailand

**Keywords:** emerging leishmaniasis, *Leishmania martiniquensis*, *Leishmania orientalis*, *Culicoides* biting midges, *Crithidia* sp., co-circulation, southern Thailand

## Abstract

Since 1996, autochthonous cases of emerging leishmaniasis caused by *Leishmania* (*Mundinia*) *martiniquensis* and *Leishmania* (*Mundinia*) *orientalis* have been more frequently reported, especially in the northern and southern parts of Thailand. However, the accurate identification of their natural vectors and reservoirs remains unconfirmed. Previous studies have suggested that these emerging parasites might be transmitted by other non-phlebotomine vectors. Herein, we speculated that *Culicoides* biting midges might act as the competent vectors responsible for autochthonous leishmaniasis in southern Thailand. In this research, 187 non-engorged, parous and gravid *Culicoides* females and 47 blood-engorged ones were trapped from the residences of two recently diagnosed visceral leishmaniasis patients in Sadao District and the unaffected site in Rattaphum District, Songkhla Province, southern Thailand. Species diversity and abundance of biting midges varied among the trapping sites. Using *ITS1*-PCR and BLASTn analysis, *L. martiniquensis* was predominantly detected in several *Culicoides* species, including *C. peregrinus*, *C. oxystoma*, *C. mahasarakhamense*, and *C. huffi* from the vicinity of patients’ houses; and in *C. fordae* and *C. fulvus* from the unaffected site. *L. orientalis* was also co-circulated in *C. peregrinus* and *C. oxystoma* caught near the second patient’s house. Additionally, *Crithidia* sp. were also detected using *SSU rRNA*-PCR across *Culicoides* spp. Host blood meal analysis of eight different *Culicoides* species from the unaffected site also revealed that all trapped *Culicoides* had fed on cows and goats, indicating the possible role of these mammalian species as reservoir hosts. Essentially, this study is the first entomological investigation, revealing the co-circulation of emerging trypanosomatids among several species of *Culicoides* biting midges and strongly supporting the potential role of this insect group as the main vectors responsible for the epidemiology of autochthonous leishmaniasis in southern Thailand.

## 1. Introduction

Leishmaniasis is a vector-borne and neglected disease commonly found in tropical and subtropical countries worldwide, impacting over one million new cases annually [1]. This disease is caused by obligatory intramacrophage parasites of the genus *Leishmania*. Until now, approximately 53 *Leishmania* species have been documented; out of those, twenty are human-pathogenic [2]. These parasites have been categorized into four subgenera, i.e., *Viannia*, *Leishmania*, *Sauroleishmania*, and the recently classified *Mundinia* (formerly known as ‘*L. enriettii* complex’) [3]. To date, five *Leishmania* species are known as members of the *Mundinia* subgenus, including *L. martiniquensis*, *L. orientalis*, *L. enriettii*, *L. macropodum*, and *L.* sp. from Ghana, increasingly impacting both human and animal health worldwide [4,5,6,7,8,9].

In the past, Thailand was regarded as a non-endemic area for leishmaniasis and only had imported cases who had returned from endemic countries [4]. However, since 1996, indigenous patients without a history of traveling abroad have been reported continuously with increasing incidence, particularly in northern and southern provinces of Thailand, indicating autochthonous transmission within the country. Over a decade, evidence of molecular characterization and phylogenetic inference has revealed that most indigenous cases previously reported in Thailand were caused by two emerging *Mundinia* species, i.e., *L. martiniquensis* and *L. orientalis*, as aforementioned [5,10].

As formerly reported, all autochthonous cases diagnosed with *L. martiniquensis* typically manifested visceral leishmaniasis (VL) and were possibly found concomitant with cutaneous leishmaniasis (CL) or mucocutaneous leishmaniasis (MCL), especially in the patients with immunosuppressive status [5,10,11,12,13,14,15,16,17,18]. Additionally, this parasite has previously been detected in cutaneous lesions of bovines in Switzerland [19] and horses in Germany and Florida [20,21]. In contrast, *L. orientalis* has been sporadically reported solely in Thailand and is mainly responsible for localized CL in immunocompetent cases and disseminated CL and VL in patients with HIV/AIDS [22,23]. Essentially, the rising prevalence of autochthonous leishmaniasis has highlighted the significance of identifying competent vectors and reservoirs of these parasites, which remain unclear [24].

In principle, phlebotomine sand flies have been widely incriminated as the natural vectors responsible for the transmission of *Leishmania* in the subgenera *Viannia*, *Leishmania*, and *Sauroleishmania* across several geographical regions of the world [25]. However, the specific identity of primary vectors of *Leishmania* species in the *Mundinia* subgenus remains obscure. Over the years, some phlebotomine species have been proposed as the potential vectors of such two autochthonous *Mundinia* species in Thailand, including *Sergentomyia khawi* [26], *Se. barraudi* [27], *Se. iyengari* [28], and *Phlebotomus stantoni* [29]. However, the natural infection of kangaroo-infecting *L. macropodum* has previously been demonstrated in day-feeding midges *Forcipomyia* spp., indicating the possible role of this insect group as the competent vectors of these *Mundinia* parasites [8].

Additionally, previous evidence revealed that *L. martiniquensis*, *L. orientalis*, and *L.* sp. from Ghana could establish infection with a high proportion of metacyclic stages in *Culicoides sonorensis* biting midges, which were subsequently able to transmit the parasites and cause the disease in BALB/c mice, supporting the vector competence of *Culicoides* biting midges [30]. More recently, *C. mahasarakhamense* specimens collected around the VL patient’s residence in Lamphun Province, northern Thailand were screened as molecularly positive for *L. martiniquensis* and avian *Trypanosoma* sp., strongly suggesting that biting midges might have an imperative role in the autochthonous transmission of these neglected pathogens [31]. However, the surveillance study showing evidence of *Culicoides* biting midges as the putative vectors for autochthonous transmission of leishmaniasis in southern Thailand has not yet been reported.

In this paper, we report two recent cases of autochthonous VL from Sadao District, Songkhla, southern Thailand during 2019–2021 and comparatively investigate the molecular prevalence of *Leishmania* and other trypanosomatids in biting midges collected around the houses of such two patients in Sadao District and from the leishmaniasis-unaffected area in Rattaphum District, Songkhla Province. Furthermore, blood meal analysis was conducted to reveal their blood-feeding preference and determine the feasibility of zoonotic transmission networks. The novel data from this research would help us better comprehend the vectorial role of *Culicoides* biting midges and the transmission biology of emerging trypanosomatids to develop the treatment, prevention, and control strategies for these neglected tropical diseases more effectively.

## 2. Materials and Methods

### 2.1. Ethics Statement

The procedures and consent of clinical specimen collection were approved by the Institutional Review Board of Hatyai Hospital (protocol number HYH EC 077-65-01). The research design and methodology for entomological and molecular investigation were approved by the animal research ethics committee of Chulalongkorn University Animal Care and Use Protocol (CU-ACUP), Faculty of Medicine, Chulalongkorn University, Bangkok, Thailand (COA No. 004/2564).

### 2.2. Patients

During 2019–2021, two local VL patients from Sadao District were clinically examined and treated by the Division of Infectious Diseases, Department of Internal Medicine, Hatyai Hospital, Songkhla Province, with their clinical course described in the Results. Clinical samples were then sent to the Center of Excellence in Vector Biology and Vector-Borne Disease, Department of Parasitology, Faculty of Medicine, Chulalongkorn University, Bangkok, Thailand, for molecular diagnosis and parasite isolation. Clinical specimen collection was performed with consent for the investigations to be a part of diagnosis and treatment. No clinical procedures in this study were conducted for research only.

### 2.3. Field Trapping of Biting Midges and Morphological Species Identification

**Figure 1:**
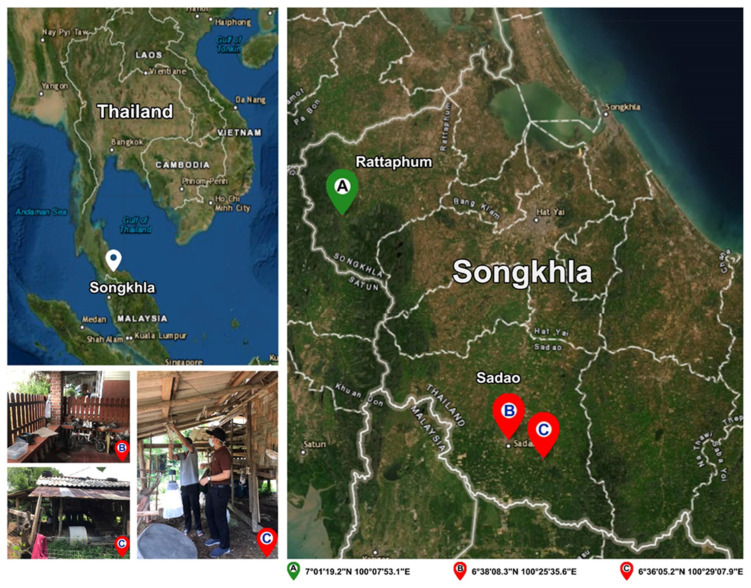
Map of specimen collection sites in this study: two VL patients’ residences in Sadao District (6°38′08.3″ N, 100°25′35.6″ E and 6°36′05.2″ N, 100°29′07.9″ E) and the unaffected area in Rattaphum District (7°01′19.2″ N, 100°07′53.1″ E), Songkhla Province, southern Thailand. The satellite map was modified from the accessible public domain (http://earthexplorer.usgs.gov/; accessed on 6 October 2022).

*Culicoides* biting midges were collected in March 2022 from the leishmaniasis-affected and non-affected areas in two districts of Songkhla Province, southern Thailand. As shown in Figure 1, the Centers for Disease Control and Prevention miniature (CDC) traps with ultraviolet lights were set up approximately 1.50 m above ground level at different locations around the houses of the two indigenous VL patients in Sadao District (6°38′08.3″ N, 100°25′35.6″ E and 6°36′05.2″ N, 100°29′07.9″ E for 1st and 2nd patients, respectively). For the non-affected area, biting midges were collected around the livestock shelters close to the jungle area, located in Rattaphum District, Songkhla Province (7°01′19.2″ N, 100°07′53.1″ E), where we also placed the traps to collect *Anopheles* mosquitoes under *Plasmodium knowlesi* malaria surveillance program. The average distance between trapping sites in the affected and non-affected areas was around 60 km.

Briefly, the traps were placed overnight from 6.00 p.m. to 6.00 a.m. for three days, and all caught specimens were anesthetized in the freezer for 30 min and then shipped in dry and frozen condition to the laboratory of the Center of Excellence in Vector Biology and Vector-Borne Disease, Department of Parasitology, Faculty of Medicine, Chulalongkorn University. Female *Culicoides* biting midges were assorted from males and other insects according to morphological characteristics such as antennae and wing patterns. Non-engorged, parous and gravid females were investigated for trypanosomatid infection, whereas nulliparous individuals, which would not have fed and would not yet have been exposed to an infectious blood meal, were discarded for analysis. Blood-engorged females were used for host blood meal analysis. For species identification, the biting midges were then identified under a stereomicroscope (SZX9, Olympus, Tokyo, Japan) using the taxonomic keys of oriental species by Wirth and Hubert [32] and the descriptions of *C. mahasarakhamense* newly recorded by Pramual et al. [33]. The intact insect cadavers were preserved individually in 70% ethanol for further DNA extraction.

### 2.4. Genomic DNA Extraction Using the Non-Destructive Enzymatic Method

To preserve the morphological structures of the specimens, genomic DNA (gDNA) was extracted from the non-engorged, parous and gravid *Culicoides* specimens and the blood-fed ones using the GenElute™ Mammalian Genomic DNA Miniprep Kit (Sigma-Aldrich, St. Louis, MO, USA) according to the non-destructive enzymatic protocol previously described by Santos et al. [34]. Briefly, to each entomological sample was added 180 µL of lysis solution C and 20 µL of the Proteinase K solution, then incubated overnight at 55 °C. The clear lysate was further extracted following the kit instructions. The obtained gDNA was eluted in a final volume of 40 µL. The gDNA concentration and purity were assessed using the NanoDrop 2000c spectrophotometer (Thermo Fisher Scientific, Waltham, MA, USA). Then, the isolated gDNA specimens were used as a template in the amplification step and kept at −20 °C for long-term storage. The remaining insect cadavers were kept dry and frozen at −20 °C until further species verification.

### 2.5. Molecular Detection of Leishmania and Other Trypanosomatids in Culicoides Biting Midge Specimens

Blood-engorged specimens were not included in pathogen screening due to the possible contamination of pathogen DNA in the host blood. Only gDNA samples extracted from non-engorged, parous and gravid biting midges were deployed for molecular screening of *Leishmania* and other trypanosomatids, including *Trypanosoma* and *Crithidia*. For *Leishmania* internal transcribed spacer 1 (*ITS1*)-specific PCR, a pair of primers: LeF (5′-TCCGCCCGAAAGTTCACCGATA-3′) and LeR (5′-CCAAGTCATCCATCGCGACACG-3′) [35] was utilized to amplify a product of approximately 379 bp, spanning over the *ITS1*, and its 5′ end of 18S and 3′ end of 5.8S ribosomal DNA flanking sequences. The PCR components were prepared in a mixture containing 50 ng of extracted gDNA, 1 µL of 10 µM each primer, 12.5 µL of 2X AccuStart™ II GelTrack PCR SuperMix (QuantaBio, Beverly, MA, USA), and nuclease-free water added to a total volume of 25 µL. The PCR thermal condition included initial denaturation at 95 °C, 5 min; 40 cycles of denaturation at 95 °C, 1 min, annealing at 65 °C, 1 min, extension at 72 °C, 1 min; and final extension at 72 °C, 10 min.

For the detection of other trypanosomatids, *SSU rRNA*-PCR was conducted using forward and reverse primers: TRY927-F (5′-GAAACAAGAAACACGGGAG-3′) and TRY927-R (5′-CTACTGGGCAGCTTGGA-3′) [36] to generate the amplicons with a size of approximately 900 bp. The PCR components were similar to those used for *ITS1*-PCR as aforementioned. The PCR thermal cycle was programmed as follows: initial denaturation of 95 °C, 5 min; 40 cycles of denaturation at 95 °C, 45 s, annealing at 53 °C, 1 min, extension at 72 °C, 1 min 30 s; and final extension at 72 °C, 10 min. The *ITS1*- and *SSU rRNA*-amplification products were separated on 1.5% (*w*/*v*) agarose gel electrophoresis with ethidium bromide staining and visualized by the Gel Doc XR+ Gel Documentation System (Bio-Rad, Hercules, CA, USA). Recombinant plasmids containing *ITS1* and *SSU rRNA* constructs and nuclease-free water were used as positive and negative controls, respectively.

### 2.6. Molecular Species Identification of Culicoides Biting Midges

To confirm morphological species identification, the representative samples of each species were selected and primarily amplified by mitochondrial *Cytochrome C oxidase subunit I* (*COI*) gene-specific PCR using the universal primers for invertebrates: LCO1490 (5′-GGTCAACAAATCATAAAGATATTGG-3′) and HCO2198 (5′- TAAACTTCAGGGTGACCAAAAAATCA-3′) [37]. If the amplification using the first primer pair was not successful, the Diptera-specific primers C1-J-1718mod (5′-GGAGGATTTGGAAATTGATTAGT-3′) and C1-N-2191mod (5′-CAGGTAAAATTAAAATATAAACTTCTGG-3′) [38] would be applied. Using 50 ng of gDNA as a template, the *COI*-PCR was programmed under the following thermal profiles: initial denaturation at 95 °C for 3 min; followed by 40 cycles of denaturation at 95 °C, 30 s; annealing at 46 °C (for LCO1490 and HCO2198) and 50 °C (for C1-J-1718mod and C1-N-2191mod), 30 s; extension at 72 °C, 1 min; and final extension at 72 °C, 10 min. The *COI* products amplified by 1st and 2nd primer pairs were visualized by 1.5% (*w*/*v*) agarose gel electrophoresis for the presence of a band of approximately 709 and 523 bp, respectively.

### 2.7. TA Plasmid Vector Cloning and Sanger Dideoxy Nucleotide Sequencing

All positive *ITS1*-, *SSU rRNA*-, and *COI*-PCR products were inserted into the pGEM^®^ T-Easy vector (Promega Corporation, Madison, WI, USA) according to the instructions. The ligation products were subsequently transformed into *Escherichia coli* DH5α competent cells and then spread onto the Luria Bertani agar plate supplemented with ampicillin, X-Gal, and IPTG for the blue/white colony screening system. Positive white clones were confirmed by colony PCR and then cultured overnight in LB broth medium with ampicillin at 37 °C. Recombinant plasmids were extracted using the Invisorb^®^ Spin Plasmid Mini Kit (STRATEC, Birkenfeld, Germany) and shipped to Macrogen, Inc. (Seoul, Republic of Korea) for bi-directional sequencing using T7 and SP6 promoter primers.

### 2.8. Sequence Alignment and Phylogenetic Analysis

Sequence chromatograms were analyzed using BioEdit Version 7.2.6 [39] for trimming the plasmid vector sequences. To reaffirm species identification of biting midges, trypanosomatid parasites, and blood meal source, the obtained sequences were then aligned with the references in GenBank by the nucleotide BLAST (BLASTn) search tool (http://blast.ncbi.nlm.nih.gov/Blast.cgi, accessed on 6 October 2022). All retrieved sequences of *Culicoides* biting midges and detected trypanosomatids were deposited in GenBank under accession numbers as listed in the Results.

For phylogenetic analyses, the obtained *ITS1* and *SSU rRNA* sequences in this study and from GenBank were gathered and analyzed by the MEGA X software [40] using the maximum likelihood (ML) method with the best-fit substitution models: Jukes–Cantor model (JC) and Tamura–Nei model with gamma distribution (TN93 + G), respectively. The bootstrap testing was conducted with 1000 bootstrap pseudoreplicates. The phylogenetic analysis of the *COI* sequences of the biting midges obtained from this study and GenBank were also built using the ML method with the general time-reversible model with gamma distribution and invariant sites (GTR + G + I) with 1000 pseudoreplicates.

### 2.9. Host Blood Meal Analysis

The extracted gDNA from each engorged individual was analyzed to identify their blood meal source using mammalian and avian *Cytochrome b* (*Cytb*)-specific PCRs. To verify mammalian DNA, UNFOR403 (5′-GGTTGTCCTCCAATTCATGTTA-3′) and UNREV1025 (5′-TGAGGACAAATATCATTCTGAGG-3′) primers [41] were applied to generate a product size of 623 bp. Likewise, screening of avian DNA was performed using degenerate primers L15557 (5′-GACTGTGACAAAATCCCNTTCCA-3′) and H16065 (5′-GGTCTTCATCTYHGGYTTACAAGAC-3′) [42] to generate an amplicon of approximately 550 bp. Both mammalian and avian *Cytb*-targeting PCR conditions include pre-denaturation at 95 °C, 5 min; followed by 40 cycles of denaturation at 95 °C, 45 s, annealing at 58 °C (for mammals) and 50 °C (for birds), 45 s, and extension at 72 °C, 1 min; and final extension at 72 °C, 10 min. The PCR products were verified by 1.5% (*w*/*v*) agarose gel electrophoresis and then subjected to direct sequencing for host species identification. The obtained chromatogram would be subsequently verified for superimposed peaks, indicative of a mixed blood-feeding pattern. The blood meal origins of engorged *Culicoides* specimens were identified using the BLASTn analysis with perfect or nearly perfect (≥98%) similarity. The bar graph showing the frequency of blood meal sources identified in each *Culicoides* species was generated using GraphPad Prism 9.4.1 (GraphPad Software Inc., San Diego, CA, USA).

## 3. Results

### 3.1. Case Presentation

#### 3.1.1. First Patient

On 9 September 2019, a 46-year-old female who lived in the urban area of Sadao District, Songkhla Province presented with prolonged fever, myalgia, fatigue, and significant weight loss for 2 months. Her underlying disease was immune thrombocytopenia, diagnosed 10 years earlier but lost to follow-up. She also had myoma uteri with hypermenorrhea and received hormonal therapy for 2 years. During 2018–2019, she had been to Saudi Arabia, Vietnam, Singapore, and Malaysia. She had lived with her relatives who came from Jordan 3 years ago. Physical examination revealed a body temperature of 37.8 °C, mild pallor, and splenomegaly. Laboratory investigations reported pancytopenia with a hemoglobin (Hb) level of 8.7 g/dL, a hematocrit of 25.3%, a white blood cell (WBC) count of 3040 cells/mm^3^ (neutrophil, 55%; lymphocyte, 33%; monocyte, 11%; atypical lymphocyte, 1%), and a platelet count of 63,000 platelets/mm^3^. Her viral serology was negative for both HIV and HCV. Ultrasonography and CT scan of the whole abdomen revealed significant splenomegaly. Histopathological examination of bone marrow biopsy exhibited erythroid hyperplasia and parasitized macrophages with intracellular amastigotes, morphologically suggestive of leishmaniasis. *Leishmania* DNA was detectable in a whole-blood sample using *Leishmania ITS1*-specific PCR. After Sanger sequencing and BLASTn search, the obtained *ITS1* sequence was identified as *L. martiniquensis* and then submitted to GenBank with assigned accession no. OP698049. The final diagnosis of VL was therefore confirmed. The patient was then administered a 1-month course of intravenous amphotericin B (1 mg/kg/d) and continued with oral itraconazole 400 mg daily for ten months. As of 1 year of follow-up, the patient was completely cured, and no relapse was observed.

#### 3.1.2. Second Patient

On 5 June 2021, a 51-year-old HIV-infected male who worked as a livestock caretaker and lived near the cattle sheds within the rubber tree plantation in Sadao District, Songkhla Province, presented with prolonged fever, anorexia, fatigue, and weight loss of 10 kg in 2 months. Five days before admission, he had persistent high-grade fever and voluminous diarrhea. He was diagnosed with HIV in 2012. He had taken an antiretroviral medication containing zidovudine, tenofovir, and lopinavir/ritonavir with low compliance, and discontinued it two months ago. His CD4 count and viral load investigated on 21 April 2021 were 26 cells/mm^3^ and 24,668 copies/mm^3^, respectively. He denied a history of overseas travel. Physical examination showed a body temperature of 38.3 °C, marked pallor, and hepatosplenomegaly. Complete blood count showed a Hb level of 8.4 g/dL, a WBC count of 1230 cell/mm^3^ (neutrophil, 62%; lymphocyte, 29%, monocyte, 8%; basophil, 1%), and a platelet count of 13,000 platelets/mm^3^, indicating pancytopenia. Abdomen ultrasonography revealed mild hepatomegaly with suspected parenchymatous disease and splenomegaly. Giemsa and Wright’s staining of the bone marrow biopsy demonstrated the presence of numerous small oval-shaped intramacrophage organisms. *Leishmania ITS1*-PCR was also positive in both whole-blood and saliva specimens. Sanger sequencing and BLASTn analysis affirmed that the detected sequence matches *L. martiniquensis*. The *ITS1* sequence was subsequently deposited to GenBank with assigned accession no. OP698050. Additionally, *Leishmania* promastigotes could be observed on day 5 after inoculating whole blood into Schneider’s insect medium supplemented with 10% fetal bovine serum. This parasite isolate was given the WHO code MHOM/TH/2021/CULE3. Based on the above investigations, he was diagnosed with VL and then treated with intravenous amphotericin B deoxycholate with a dose of 1 mg/kg/d for 5 weeks, followed by 400 mg of oral itraconazole daily for one month. However, he lost to follow-up after a month. In October 2021, his CD4 count decreased to 7 cells/mm^3^ and his viral load increased to 72,853 copies/mm^3^. In March 2022, he was readmitted with prolonged fever, recurrent voluminous diarrhea, and a urinary tract infection. The examination also revealed hepatosplenomegaly and pancytopenia, suspicious of recurrent VL. Empirical antibiotics and amphotericin B were administered; however, the patient did not respond well, then progressed to acute renal failure and febrile neutropenia. Finally, he died of septic shock resulting from the nosocomial infection of multidrug-resistant *Acinetobacter baumannii*.

### 3.2. Morphological Identification and COI-Based Species Confirmation of Field-Caught Culicoides Biting Midges

**Figure 2:**
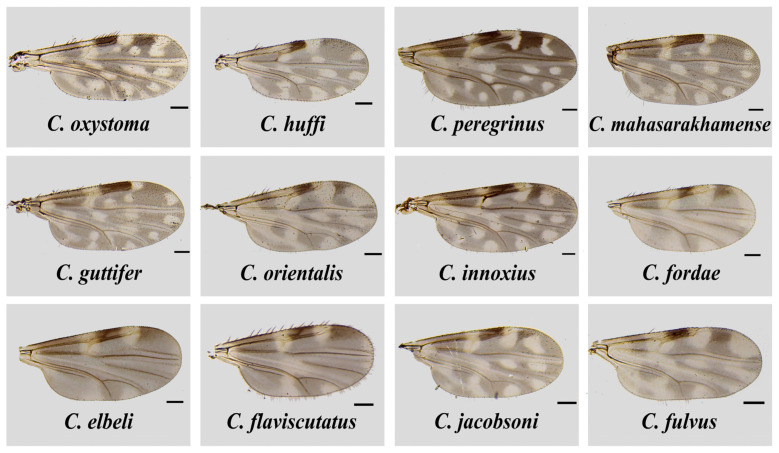
Wing characteristics of *Culicoides* species identified from the three trapping sites in Sadao and Rattaphum Districts, Songkhla Province, in this study. The scale bar represents 100 µm.

The total of 187 non-engorged, parous and gravid biting midges consisted of 91 females collected from the house surroundings of two local VL patients in Sadao District as aforementioned, and 96 individuals randomly sampled from hundreds of biting midges trapped in the unaffected site in Rattaphum District. Only 47 engorged specimens were caught solely from the unaffected site for host blood meal analysis. *Culicoides* samples were morphologically identified as thirteen distinctive species in five subgenera, i.e., *Remmia*, *Hoffmania*, *Meijerehelea*, *Avaritia*, and *Trithecoides*, as well as the *Calvipalpis* group, as detailed in Table 1. As demonstrated in Figure 2, the characteristic wing patterns were mainly deployed as the key morphological features for species identification. In the area of leishmaniasis, non-engorged *C. oxystoma* (*n* = 11, 44%) and *C. peregrinus* (*n* = 34, 51.5%) were the most abundant species found near the residences of the first and second patients, respectively. In contrast, *Trithecoides* members, including *C. fordae*, *C. elbeli*, *C. flaviscutatus*, and *C. Trithecoides* sp., were found as the most common group (*n* = 83, 86.5%, and *n* = 33, 70.2% for non-engorged and blood-fed females, respectively) in the leishmaniasis-unaffected setting in Rattaphum District.

**Figure 3:**
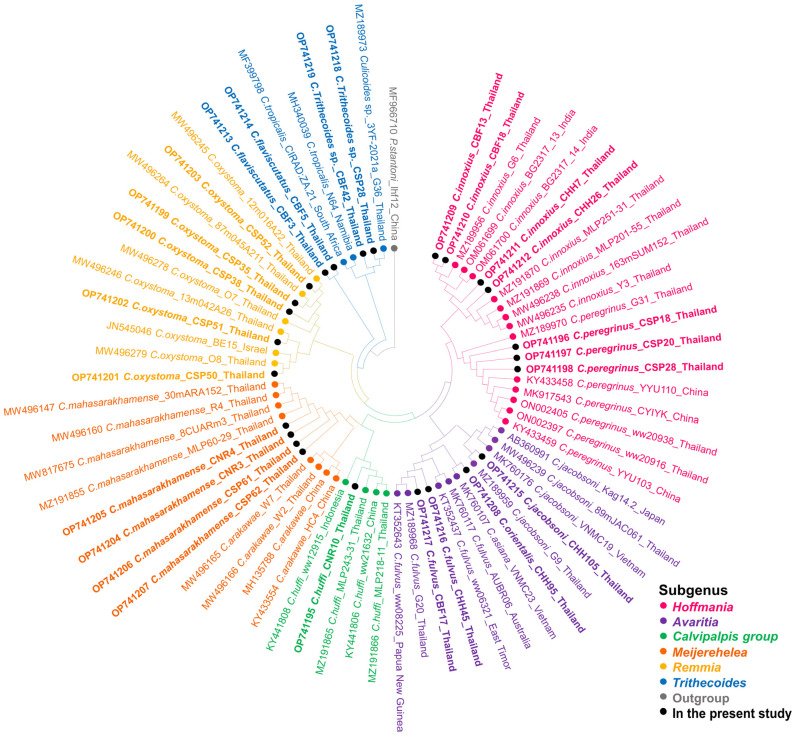
Species reaffirmation of *Culicoides* biting midges according to the *COI* phylogeny using the maximum likelihood method with the GTR + G + I model. Different colors represent the subgenera of *Culicoides*: *Hoffmania* (pink), *Avaritia* (purple), *Calvipalpis* group (green), *Meijerehelea* (orange), *Remmia* (yellow), and *Trithecoides* (blue). The *Culicoides* species identified in the present study are labeled with solid black circles. *Ph. stantoni* was used as the outgroup and marked with gray.

For *COI*-PCR, only samples of *C. peregrinus* and *C. flaviscutatus* were successfully amplified using the LCO1490 and HCO2198 primer pair. More satisfactorily, the Diptera-specific primers, C1-J-1718mod and C1-N-2191mod, gave positive results for most other *Culicoides* species. Unfortunately, no successful amplification and sequencing were obtained from specimens of *C. guttifer*, *C. fordae*, and *C. elbeli* collected in this study. The twenty-five *COI* sequences were annotated and deposited in GenBank with assigned accession numbers OP741195-OP741219 as detailed in Table S1. Almost all obtained sequences (22/25) matched with conspecific references available in GenBank with high similarity percentages (96.20–100%), indicating that our morphological identification was correct. The lower similarity scores were observed in three specimens of *C. flaviscutatus* and *C. Trithecoides* sp., due to the unavailability of their conspecific references in GenBank. To support the BLASTn result, the ML phylogenetic tree of sixty-nine sequences from this study and those from GenBank was constructed, clearly indicating that our identified species were distinctively placed with their conspecific references, as demonstrated in Figure 3.

### 3.3. Molecular Prevalence and Phylogenetic Analysis of Leishmania and Crithidia in Non-Engorged Culicoides Biting Midges

**Figure 4:**
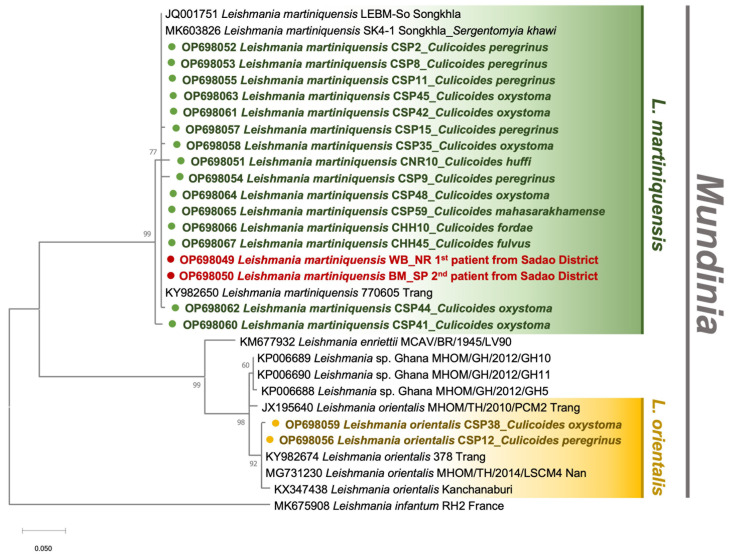
Maximum likelihood phylogenetic tree of the *Leishmania ITS1* sequences retrieved from *Culicoides* biting midges in this study and Genbank, using the JC model. The colored circles indicate our sequences, including *L. martiniquensis* from six species of *Culicoides* biting midges (green) and the two recently diagnosed patients (red), and *L. orientalis* from *C. oxystoma* and *C. peregrinus* (yellow). *L. infantum* isolate RH2 was used as the outgroup.

The gDNA specimens isolated from a collection of 187 non-engorged, parous and gravid *Culicoides* spp. were screened for *Leishmania* and other trypanosomatids, using *ITS1*- and *SSU rRNA*-specific PCRs, respectively. The field infection rate was calculated by the ratio of positive samples to the total individuals collected in each locale. In the study area of leishmaniasis, *C. huffi* (*n* = 1, 4%) as well as *C. peregrinus* (*n* = 6, 9.1%), *C. oxystoma* (*n* = 7, 10.6%), and *C. mahasarakhamense* (*n* = 1, 1.5%) from the residences of the first and second patients, respectively, tested positive for *Leishmania* DNA, as informed in Table 1. Surprisingly, *Leishmania* DNA was also amplified in two individuals of *C. fordae* and *C. fulvus* caught from the leishmaniasis-unaffected area. As listed in Table S2, the BLASTn analysis revealed that out of 19 sequences, seventeen matched with the *ITS1* references of *L. martiniquensis* (accession numbers KY982650 and MK603826) previously reported in GenBank with a similarity score of 98–100%. The other two sequences from two individuals of *C. peregrinus*, and *C. oxystoma* collected from the second patient’s house were significantly identical to the *L. orientalis* 378_Trang reference (accession no. KY982674). The *Leishmania ITS1* sequences obtained from our *Culicoides* specimen collection were submitted to the GenBank database and assigned the accession numbers OP698051-OP698067. More importantly, phylogenetic analysis revealed a cluster of *L. martiniquensis* sequences obtained from *Culicoides* samples and our two VL patients and showed a clear divergence between *L. martiniquensis* and *L. orientalis*, as demonstrated in Figure 4.

**Figure 5:**
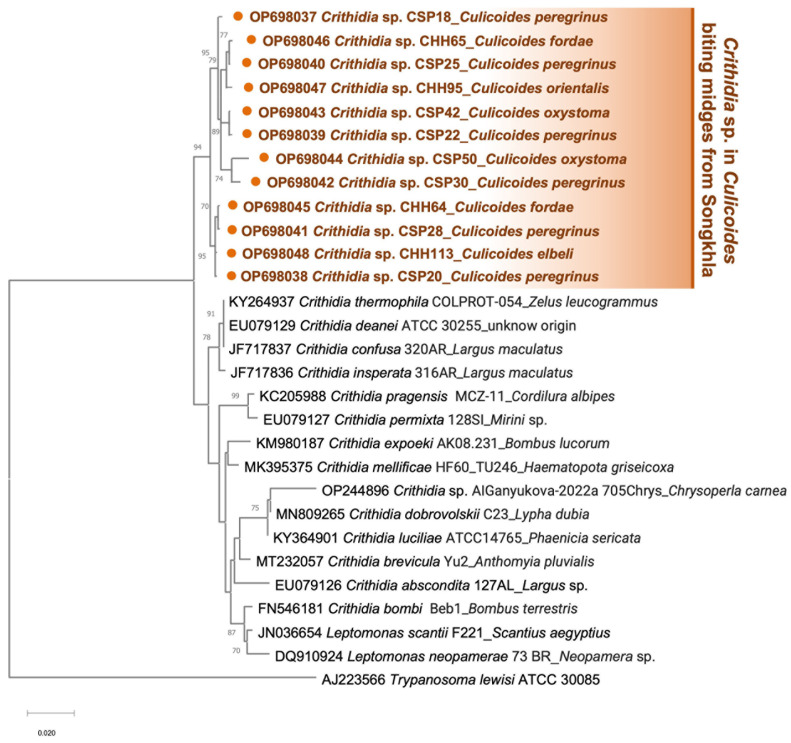
Maximum likelihood phylogenetic tree of the *Crithidia SSU rRNA* sequences retrieved from *Culicoides* biting midges in this study and Genbank, using the TN93 + G model. The solid orange circles indicate our sequences, including *Crithidia* sp. from six *Culicoides* species. *T. lewisi* ATCC 30085 was used as the outgroup.

For *SSU rRNA* amplification, *C. peregrinus* (*n* = 6, 9.1%), and *C. oxystoma* (*n* = 2, 3%) from the second patient’s house, and specimens of *C. fordae* (*n* = 2, 2.1%), *C. elbeli* (*n* = 1, 1%) and *C. orientalis* (*n* = 1, 1%) from the unaffected site were detected positive. As detailed in Table S3, the BLASTn alignment also showed that all amplified *SSU rRNA* sequences significantly matched with *C. thermophila*/*confusa*/*deanei* reference (accession numbers KY264937/JF717837/EU079129). However, no samples in this investigation tested positive for *Trypanosoma*. Then, all obtained sequences were identified as *Crithidia* sp. and uploaded to GenBank with assigned accession numbers OP698037-OP698048. Surprisingly, a specimen of *C. oxystoma* (CSP42) from the second patient’s house was also found to be co-infected with both *L. martiniquensis* and *Crithidia* sp. Phylogenetic inference showed a cluster of *Crithidia* sp. amplified in this study, obviously discriminating from other *Crithidia* spp., as depicted in Figure 5.

### 3.4. Host Blood Meal Identification

**Figure 6:**
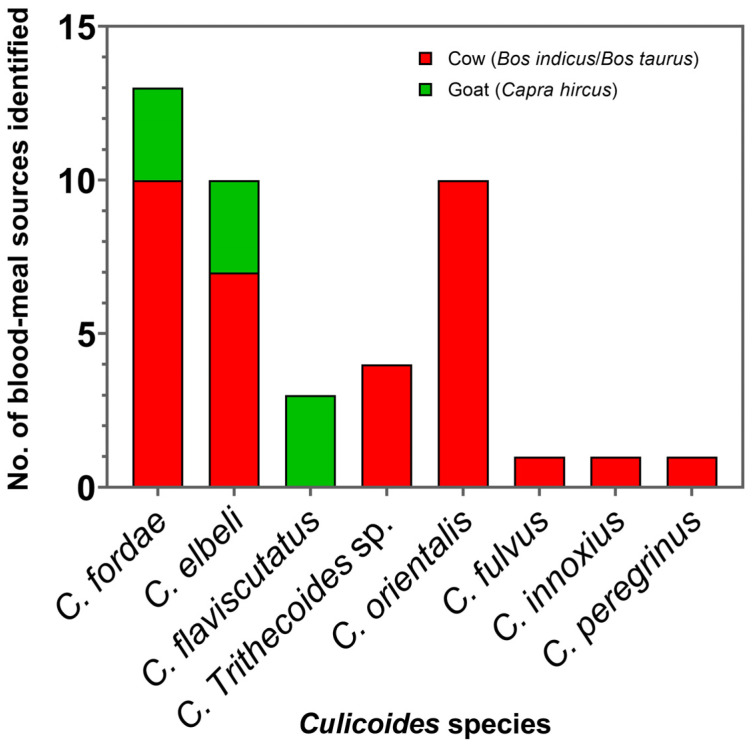
Bar graph revealing the number of blood meal origins detected in *Culicoides* biting midges collected near the livestock sheds in Rattaphum District, Songkhla Province.

In this study, 47 blood-engorged Culicoides females, consisting of the members in the subgenera *Trithecoides*; *C. fordae*, *C. elbeli*, *C. flaviscutatus*, and *C. Trithecoides* sp.), *Avaritia* (*C. orientalis* and *C. fulvus*), and *Hoffmania* (*C. innoxius* and *C. peregrinus*), were confined only to the non-affected site in Rattaphum District as detailed in Table 1. Of these tested, only 43 specimens consisting of *C. fordae* (*n* = 13, 30.2%), *C. elbeli* (*n* = 10, 23.3%), *C. flaviscutatus* (*n* = 3, 7%), *C. Trithecoides* sp. (*n* = 4, 9.3%), *C. orientalis* (*n* = 10, 23.3%), *C. fulvus* (*n* = 1, 2.3%), *C. innoxius* (*n* = 1, 2.3%), and *C. peregrinus* (*n* = 1, 2.3%) were screened positive using mammalian-specific UNFOR403 and UNREV1025 primers. Based on the BLASTn alignment, only two mammalian species, including cow (*Bos indicus*/*Bos taurus*, *n* = 34, 79%) and goat (*Capra hircus*, *n* = 9, 21%), were identified as hosts. For avian DNA detection, 37 specimens were successfully amplified using degenerate L15557 and H16065 primers. However, none were detected positive for the avian hosts since all obtained sequences matched ≥98% with the same mammalian references as those using mammal-specific primers. This cross-amplification might be due to the low specificity of this pair of degenerate primers under the PCR condition used in this work. Additionally, no superimposed peaks were observed after chromatogram verification, indicating no multiple blood-feeding patterns in these engorged specimens. As revealed in Figure 6, *C. orientalis*, *C. Trithecoides* sp., *C. fulvus*, *C. peregrinus*, and *C. innoxius* fed solely on cows, whereas *C. flaviscutatus* preferred goats as hosts. In addition, opportunistic feeding behavior was observed in *C. fordae*, and *C. elbeli*, preferably acquiring blood meals primarily from cows over goats.

## 4. Discussion

Here, we investigated the diversity and abundance of *Culicoides* biting midge fauna and molecular prevalence of *Leishmania* and other trypanosomatids among the collection sites in Songkhla Province, southern Thailand, to assess the vector potential of *Culicoides* for autochthonous transmission of leishmaniasis. In the present study, variations in species compositions and abundance were observed among the trapping sites. Most *Culicoides* specimens, comprising 61.1% of all tested individuals, were caught from the livestock sheds in Rattaphum District, whereas the first patient’s house provided the lowest number of samples, accounting for only 10.7%. Importantly, several factors, including ecological habitats, seasonality, host availability, wind speed, trap height, and distances between traps and animal lures, may have influenced the abundance of *Culicoides* species among these trapping sites [43,44,45,46,47]. It could be explained that the first patient’s house was located in a city area with a decline of trees; therefore, *Culicoides* could be more prone to be dispersed away from the traps by wind, whereas other collection sites were close to the plantation or forest, sheltering the midges from the wind and resulting in a larger collection. In addition, trap placement adjacent to the livestock sheds especially yielded much larger catches. The soil environment is also another major factor influencing *Culicoides* abundance, as biting midges need optimal soil substrates for their larval development and adult emergence [43,48,49].

Previously, several publications have reported the positive detection of *Leishmania* parasites in other subgenera, including *Leishmania* (*Leishmania*) *infantum* [50], *Leishmania* (*Leishmania*) *mexicana* [51], *Leishmania* (*Leishmania*) *amazonensis* [52], and *Leishmania* (*Viannia*) *braziliensis* [52] in different species of *Culicoides* biting midges. It is, therefore, reasonable to hypothesize that *Culicoides* biting midges potentially serve as hosts of *Leishmania* parasites in the subgenus *Mundinia* as well.

In this study, nulliparous specimens, which typically dominate collections, were not included in pathogen screening because this physiological stage had never fed, and therefore would not have been exposed to any infectious blood meal from the hosts [53]. For blood-engorged samples, parasite detection is feasible; however, we cannot ascertain the parasite infection in the insects using this type of specimen because the parasite detection could be positive, possibly due to host blood contamination with parasite DNA. Differently, non-engorged, parous and gravid *Culicoides* individuals, which had entered the gonotrophic cycle by feeding on the hosts for their oviposition, would be expected to have been exposed to and acquired parasites from an infectious blood meal [53,54]. Accordingly, these two types of specimens, parous and gravid, are appropriate for molecular screening to indicate the parasite infection in this investigation.

We found that *L. martiniquensis* was detected positive only in one female of *C. huffi* from the house surroundings of the first patient who was cured in 2020 and was not an active case at the time of midge collection. This low prevalence ratio could be ascribed to the limited number of collected specimens and the unavailability of animal reservoirs in the vicinity of the patient’s place. More importantly, a higher prevalence of *L. martiniquensis* was observed in 18.2% (12/66) of non-engorged females collected around the second patient’s house, including *C. peregrinus*, the most abundant species, followed by *C. oxystoma*, and *C. mahasarakhamense*. This evidence corroborates the previous report by Collins et al. [43] claiming that the most abundant *Culicoides* species identified were the potential vectors of bluetongue virus and Schmallenberg virus in northern Europe. It can be implied that dominant *Culicoides* species tend to serve as the putative vectors as they are more likely to enhance the transmission of pathogens. Our results were also consistent with the recent study by Sunantaraporn et al. [31] that proposed *C. mahasarakhamense* as one of the potential vectors of *L. martiniquensis* in northern Thailand. 

Besides *L. martiniquensis*, *L. orientalis* has been detected for the first time in two specimens of *C. peregrinus* and *C. oxystoma*, indicating the co-circulation of these emerging parasites in the same area. It was also inferred that these two *Mundinia* species could share the same vector species and potentially harbor the same animal reservoirs on which such vectors feed. 

Of note, the overall prevalence of *Leishmania*-infected midges in this transmission area was 21.2% (14/66), higher than that previously reported in sand flies *Se. khawi* (5.41%) [26] and *Ph. stantoni* (12.5%) [29] collected from Songkhla and Chiang Rai Provinces, respectively. Accordingly, this high detection frequency was possibly attributed to our prompt field surveillance once the diagnosis was established. As aforementioned, *L. martiniquensis* has previously been reported in peridomestic animals, namely bovines in Europe [19] and horses in Europe and the USA [20,21]. In Thailand, Kongkaew et al. [55] reported seropositivity for *Leishmania* antibodies in three cows and one cat (*Felis catus*) investigated near the VL patient’s house in Nan Province, northern Thailand. Recently, Sriwongpan et al. [29] revealed the presence of *Leishmania* antibodies in two water buffaloes (*Bubalus bubalis*), two dogs (*Canis familiaris*), and one black rat (*Rattus rattus*) from the leishmaniasis-affected area in Chiang Rai Province, northern Thailand. Furthermore, *L. martiniquensis* DNA was detected in the blood, liver, and spleen tissues of two black rats captured from the VL patient’s residence in Na Thawi District, Songkhla Province [27], and in the buffy coat of a black rat from the patient’s house in Chiang Rai Province [29]. As aforementioned, several pieces of serological and molecular evidence for the circulation of *Leishmania* have strongly implicated the peridomestic animals as putative reservoirs of these autochthonous parasites. From the clinical history known, this second patient worked as a cattle caretaker; therefore, it was feasible that livestock potentially serves as the parasite reservoirs in this affected area.

In addition, the positive detection of *L. martiniquensis* was also observed in *C. fordae* and *C. fulvus* individuals caught from the unaffected site. We speculated that these infected midges might be spread over long distances from the affected area by the prevailing wind [56,57,58,59]. Moreover, our blood meal analysis also confirmed that the putative vectors, *C. peregrinus*, *C. fordae*, and *C. fulvus*, have a blood-feeding preference on cows, suggesting that the parasites had probably been circulating between vectors and reservoirs even in the area where clinical cases had never been reported.

Importantly, *L. martiniquensis ITS1* sequences obtained from our collected *Culicoides* were phylogenetically similar to those of two VL cases reported in this study. This sympatric occurrence suggests that *Culicoides* biting midges most likely involve the autochthonous transmission of *Leishmania* parasites in these two localities. In addition, our *ITS1* sequences derived from *Culicoides* spp. were closely clustered into the same clade as that amplified from *Se. khawi* previously collected from Na Thawi District, Songkhla Province (accession no. MK603826) [26], and the HIV/AIDS patient from Trang Province (accession no. KY982650) [60] as formerly reported. Therefore, it could be inferred that this *Mundinia* parasite can infect a broad range of insect hosts, including sand flies and biting midges, and has been distributed widely in other localities apart from Songkhla Province.

In this study, blood meal source identification revealed a mammalophilic preference of all collected blood-engorged *Culicoides* spp., feeding on cows and goats, as the light traps were placed near the livestock shelters. It was also shown that two species, namely *C. fordae* and *C. elbeli*, opportunistically fed on both two hosts, possibly determined by host availability around the trapping sites. Surprisingly, in this study, none tested positive for humans, possibly due to a limited number of engorged biting midges trapped near the livestock sheds only from the unaffected location. A previous study of Thai *Culicoides* by Jomkumsing et al. [61] revealed that *C. peregrinus* and *C. oxystoma* fed on cattle, whereas *C. mahasarakhamense*, *C. huffi*, and *C. fulvus* attacked domestic chickens. To support *Culicoides* as the suspected vectors of human leishmaniasis, the evidence of feeding on humans needs to be demonstrated [62]. To our knowledge, feeding on humans by *Culicoides* species in Thailand has never been recorded thus far.

Recently, some investigators from West Bengal, India demonstrated that three *Culicoides* species, namely *C. peregrinus*, *C. oxystoma*, and *C. huffi*, were found feeding opportunistically on humans, implicating them as the putative vectors for the anthropozoonotic transmission of pathogens, including *Leishmania* parasites [63]. This finding also implies that such *Culicoides* species, suspected vectors of leishmaniasis in our country, could also feed opportunistically on humans. Therefore, a more extensive collection of engorged specimens, especially from different affected sites, is needed for further blood source analysis to elucidate this speculation.

Our molecular evidence of parasite DNA in the insect specimens supported that the positive *Culicoides* species can serve as potential or putative vectors of leishmaniasis. However, it was insufficient to indicate those as proven vectors responsible for the transmission cycle of leishmaniasis in the natural setting. In *Leishmania*, parasite development is a complex process that might be prevented through physical barriers and innate immunity, especially in unpermissive insect species [64,65,66]. These natural barriers, including secreted proteolytic enzymes, peritrophic matrix, and insect immune reactions, may reduce parasite numbers, prevent parasite escape from the remnants of digested blood meal or even inhibit late-stage parasite development, respectively [64,65,66]. In this case, the parasite DNA was possibly detectable, but transmission cannot occur.

Most importantly, the successful development of infective metacyclic promastigotes and the capability of infected *Culicoides* to transmit the infective parasites need to be demonstrated to confirm the vector competence of this insect group. As previously published, Becvar et al. [30] revealed the success of infection, metacyclic development, and transmission of three *Mundinia* species, namely *L. martiniquensis*, *L. orientalis*, and *L*. sp. from Ghana, in the biting midge *C. sonorensis* under experimental conditions. This evidence strongly suggests *Culicoides* as a competent vector for leishmaniasis. In addition, it was also experimentally demonstrated that all *Mundinia* species exhibit suprapylarian development with the colonization of the stomodeal valve and the part of thoracic midgut with late-stage parasites [30,67]. To prove the vector competence of Thai *Culicoides* species, further studies of experimental transmission or investigation of parasite development and localization within the insects are required.

Furthermore, *Crithidia* sp. was molecularly screened in several biting midge species, primarily prevalent in *C. peregrinus* in this study. Unlike *Leishmania*, this parasite has a single-host (monoxenous) life cycle and typically parasitizes in a digestive tract of a wide variety of insect groups from the orders Diptera, Hemiptera, Hymenoptera, and Siphonaptera worldwide [68,69,70]. Interestingly, our *Crithidia* sequences were phylogenetically grouped into a distinctive clade with high bootstrap support, obviously separating from other *Crithidia* species detected in other insect groups. This phylogenetic divergence implies that *Culicoides*-infecting *Crithidia* identified in this study is likely to be a novel species. Our finding also reaffirmed the previous investigation by Sunantaraporn et al. [71], revealing that *Crithidia* sp. was detected in two individuals of *C. peregrinus* collected from the VL patient’s residence in the neighboring Phatthalung Province; however, none of those tested positive for *Leishmania* DNA. Although the monoxenous trypanosomatids are generally considered non-pathogenic, the opportunistic infection of these parasites, including *Crithidia,* has been sporadically reported in both humans and other vertebrate hosts [72,73,74,75,76,77].

As previously reported by Ghobakhloo et al. [74], *Crithidia* infection and co-infection with *L. major* were found in some cases with suspected CL from Iran. In addition, the clinical isolates of *Crithidia* sp. in that study could survive at 37 °C and establish the infection in two macrophage cell lines (THP1 and J774), indicating its high potential to cause *Leishmania*-like pathogenesis in humans and other mammals. However, Kostygov et al. [78] have proposed that the ability of monoxenous species to tolerate the increased temperature and the host’s immunosuppressive status are considered essential factors for disease development. In the current study, we found the co-circulation of *Crithidia* sp. and *Leishmania* spp. in *Culicoides* individuals collected from the same locations. Moreover, *L. martiniquensis* and *Crithidia* sp. co-infection was observed in a single sample of *C. oxystoma* (CSP42). Although atypical *Crithidia* infection has never been clinically reported in Thailand, our results suggest the possible transmission of *Crithidia* parasites to humans, particularly in the leishmaniasis-affected areas of the country. However, *Crithidia* remains far from understood, and more surveillance and investigations are needed to confirm the pathogenicity of this presumably monoxenous parasite.

Ultimately, we can infer from our molecular findings that *Culicoides* biting midges are most likely to be competent vectors of emerging leishmaniasis, especially in southern Thailand. Moreover, the evidence of co-circulation of *Leishmania* and *Crithidia* in *Culicoides* has emphasized the complexity of autochthonous transmission and disease epidemiology in the country. Essentially, further experiments for incriminating *Culicoides* as the natural vectors and investigations of reservoir hosts are required to comprehend the transmission biology of trypanosomatids better and establish the prevention and control strategies more effectively.

## 5. Conclusions

The increasing prevalence of autochthonous leishmaniasis in Thailand has emphasized the importance of incriminating principal vectors of *Leishmania* parasites. Recent publications have proposed that certain *Leishmania* species can develop in biting midge *Culicoides sonorensis*, suggesting the possible role of *Culicoides* in disease transmission. In this research, we report the two recently diagnosed VL cases as well as demonstrate the first molecular evidence of predominant *L. martiniquensis* in different species of biting midges, including *C. peregrinus*, *C. oxystoma*, *C. mahasarakhamense*, *C. huffi*, *C. fordae*, and *C. fulvus*; and *L. orientalis* in *C. peregrinus*, and *C. oxystoma*, collected from Songkhla Province, southern Thailand. In addition, *Crithidia* sp., a monoxenous trypanosomatid, was also detected in *C. peregrinus*, *C. oxystoma*, *C. fordae*, *C. elbeli*, and *C. orientalis*. Furthermore, blood meal analysis indicated a mammalophilic behavior of *Culicoides* collected in this study, suggesting the possible parasite reservoirs and the risk of zoonotic transmission. Essentially, this novel finding reveals the molecular evidence of parasite co-circulation and supports that *Culicoides* biting midges potentially serve as the competent vectors of these emerging *Mundinia* parasites in southern Thailand.

## Figures and Tables

**Table 1:** Species diversity, relative abundance of *Culicoides* biting midges, and molecular detection of trypanosomatid parasites across the three sampling locations in this study.

Collection Sites	Subgenus	Host Species	No. of Investigated Biting Midges			Sample ID. of Non-Engorged Biting Midges with Positive PCR	
			Male (*n* = 19)	Non-Engorged (Parous and Gravid) Female (*n* = 187)	Engorged (*n* = 47)	*ITS1*-PCR (*n* = 17)	*SSU rRNA*-PCR (*n* = 12)
Sadao District, Songkhla Province 1st Patient’s House (6°38′08.3″ N, 100°25′35.6″ E)	*Remmia*	*C. oxystoma*	3	11			
	*Calvipalpis* group	*C. huffi*		7		CNR10	
	*Hoffmania*	*C. peregrinus*	5				
	*Meijerehelea*	*C. mahasarakhamense*	1	4			
	*Meijerehelea*	*C. guttifer*		1			
	*Avaritia*	*C. orientalis*		1			
	*Trithecoides*	*C. Trithecoides* sp.		1			
		Total	9	25	0	1	0
Sadao District, Songkhla Province 2nd Patient’s House (6°36′05.2″ N, 100°29′07.9″ E)	*Hoffmania*	*C. peregrinus*	9	34		CSP2, CSP8, CSP9, CSP11, CSP12, CSP15	CSP18, CSP20, CSP22, CSP25, CSP28, CSP30
	*Hoffmania*	*C. innoxius*		2			
	*Remmia*	*C. oxystoma*	1	22		CSP35, CSP38, CSP41, CSP42 *, CSP44, CSP45, CSP48	CSP42 *, CSP50
	*Meijerehelea*	*C. mahasarakhamense*		6		CSP59	
	*Meijerehelea*	*C. guttifer*		1			
	*Avaritia*	*C. orientalis*		1			
		Total	10	66	0	14	8
Rattaphum District, Songkhla Province Livestock Sheds (7°01′19.2″ N, 100°07′53.1″ E)	*Trithecoides*	*C. fordae*		45	15	CHH10	CHH64, CHH65
	*Trithecoides*	*C. elbeli*		28	11		CHH113
	*Trithecoides*	*C. flaviscutatus*			3		
	*Trithecoides*	*C. Trithecoides* sp.		10	4		
	*Avaritia*	*C. orientalis*		5	11		CHH95
	*Avaritia*	*C. fulvus*		2	1	CHH45	
	*Avaritia*	*C. jacobsoni*		2			
	*Hoffmania*	*C. innoxius*		3	1		
	*Hoffmania*	*C. peregrinus*			1		
	*Meijerehelea*	*C. guttifer*		1			
		Total	0	96	47	2	4

‘*’, a single specimen of *C. oxystoma* (CSP42) was co-infected by *L. martiniquensis* and *Crithidia* sp.

## Data Availability

All data generated or analyzed during this study are included in this published article and its Supplementary Information Files.

## Supplementary Materials

The following supporting information can be downloaded at: https://www.mdpi.com/2414-6366/7/11/379/s1, Table S1: The BLASTn result of *COI* sequences obtained from representative samples of *Culicoides* species in this study; Table S2: The BLASTn result of *Leishmania ITS1* sequences amplified from field-caught *Culicoides* biting midges in this study; Table S3: The BLASTn result of *Crithidia SSU rRNA* sequences derived from field-caught *Culicoides* biting midges in this study.
