# Supplementary material for: First Evidence of Co-Circulation of Emerging Leishmania martiniquensis, Leishmania orientalis, and Crithidia sp. in Culicoides Biting Midges (Diptera: Ceratopogonidae), the Putative Vectors for Autochthonous Transmission in Southern Thailand
Source: Trop Med Infect Dis. 2022 Nov 15;7(11):379. doi: 10.3390/tropicalmed7110379 (PMC9696774; doi:10.3390/tropicalmed7110379)
Supplement: Supplementary file 1 [file tropicalmed-07-00379-s001.zip › tropicalmed-2043638-supplementary.pdf]

Tropical Medicine and Infectious Disease

MDPI

Supplementary Materials for First Evidence of Co-Circulation of Emerging *Leishmania martiniquensis*, *Leishmania orientalis*, and *Crithidia* sp. in *Culicoides* Biting Midges (Diptera: Ceratopogonidae), the Putative Vectors for Autochthonous Transmission in Southern Thailand

Table S1. The BLASTn result of COI sequences obtained from representative samples of Culicoides Species in this study.

No.	Sample ID	Accession No.	Morphological Identification	Top BLASTn Hits and % Similarity
1.	CNR10	OP741195	*C. huffi*	*C. huffi* MLP218-11 (MZ191866), 96.20%
2.	CSP18	OP741196	*C. peregrinus*	*C. peregrinus* YYU_103 (KY433459), 99.52%
3.	CSP20	OP741197	*C. peregrinus*	*C. peregrinus* YYU_103 (KY433459), 100%
4.	CSP28	OP741198	*C. peregrinus*	*C. peregrinus* YYU_103 (KY433459), 100%
5.	CSP35	OP741199	*C. oxystoma*	*C. oxystoma* 87m045A211 (MW496264), 99.28%
6.	CSP38	OP741200	*C. oxystoma*	*C. oxystoma* O7 (MW496278), 99.52%
7.	CSP50	OP741201	*C. oxystoma*	*C. oxystoma* O8 (MW496279), 100%
8.	CSP51	OP741202	*C. oxystoma*	*C. oxystoma* 13m042A26 (MW496246), 99.05%
9.	CSP52	OP741203	*C. oxystoma*	*C. oxystoma* 12m016A22 (MW496245), 99.29%
10.	CNR3	OP741204	*C. mahasarakhamense*	*C. mahasarakhamense* MLP60-29 (MZ191855), 100%
11.	CNR4	OP741205	*C. mahasarakhamense*	*C. mahasarakhamense* MLP60-29 (MZ191855), 100%
12.	CSP61	OP741206	*C. mahasarakhamense*	*C. mahasarakhamense* MLP60-29 (MZ191855), 99.76%
13.	CSP62	OP741207	*C. mahasarakhamense*	*C. mahasarakhamense* MLP60-29 (MZ191855), 99.76%
14.	CHH95	OP741208	*C. orientalis*	*C. asiana* VNMC23 (MK760107), 99.76%
15.	CBF13	OP741209	*C. innoxius*	*C. innoxius* G6 (MZ189956), 99.76%
16.	CBF18	OP741210	*C. innoxius*	*C. innoxius* G6 (MZ189956), 99.76%
17.	CHH7	OP741211	*C. innoxius*	*C. innoxius* G6 (MZ189956), 99.27%
18.	CHH26	OP741212	*C. innoxius*	*C. innoxius* MLP251-31 (MZ191870), 100%
19.	CBF3	OP741213	*C. flaviscutatus*	*C. sinanoensis* RUXX03 (MK760243), 87%
20.	CBF5	OP741214	*C. flaviscutatus*	*C. sinanoensis* RUXX03 (MK760243), 87%
21.	CHH105	OP741215	*C. jacobsoni*	*C. jacobsoni* G9 (MK189959), 100%
22.	CHH45	OP741216	*C. fulvus*	*C. fulvus* ww08225 (KT352643), 100%
23.	CBF17	OP741217	*C. fulvus*	*C. fulvus* ww08225 (KT352643), 100%
24.	CBF28	OP741218	*C. Trithecoides* sp.	*Culicoides* sp. 3 YF-2021a G36 (MZ189973), 100%
25.	CBF42	OP741219	*C. Trithecoides* sp.	*C. tropicalis* CIRAD:ZA.21 (MF399798), 85.20%

Table S2. The BLASTn result of *Leishmania ITS1* sequences amplified from field-caught *Culicoides* biting midges in this study.

No.	Location	Accession no.	Parasite Identification	Isolate	Host Species	Top BLASTn Matches and % Similarity
1.	Sadao/1st patient house	OP698051	*L. martiniquensis*	CNR10	*C. huffi*	*L. martiniquensis* 770605_Trang (KY982650), 98.84%
2.	Sadao/2nd patient house	OP698052	*L. martiniquensis*	CSP2	*C. peregrinus*	*L. martiniquensis* 770605_Trang (KY982650), 100%
3.	Sadao/2nd patient house	OP698053	*L. martiniquensis*	CSP8	*C. peregrinus*	*L. martiniquensis* 770605_Trang (KY982650), 100%

4.	Sadao/2nd patient house	OP698054	*L. martiniquensis*	CSP9	*C. peregrinus*	*L. martiniquensis* 770605_Trang (KY982650), 98.84%
5.	Sadao/2nd patient house	OP698055	*L. martiniquensis*	CSP11	*C. peregrinus*	*L. martiniquensis* 770605_Trang (KY982650), 99.61%
6.	Sadao/2nd patient house	OP698056	*L. orientalis*	CSP12	*C. peregrinus*	*L. orientalis* 378_Trang (KY982674), 100%
7.	Sadao/2nd patient house	OP698057	*L. martiniquensis*	CSP15	*C. peregrinus*	*L. martiniquensis* 770605_Trang (KY982650), 99.61%
8.	Sadao/2nd patient house	OP698058	*L. martiniquensis*	CSP35	*C. oxystoma*	*L. martiniquensis* SK4-1 (MK603826), 99.61%
9.	Sadao/2nd patient house	OP698059	*L. orientalis*	CSP38	*C. oxystoma*	*L. orientalis* 378_Trang (KY982674), 98%
10.	Sadao/2nd patient house	OP698060	*L. martiniquensis*	CSP41	*C. oxystoma*	*L. martiniquensis* 770605_Trang (KY982650), 98.84%
11.	Sadao/2nd patient house	OP698061	*L. martiniquensis*	CSP42	*C. oxystoma*	*L. martiniquensis* 770605_Trang (KY982650), 99.61%
12.	Sadao/2nd patient house	OP698062	*L. martiniquensis*	CSP44	*C. oxystoma*	*L. martiniquensis* 770605_Trang (KY982650), 99.61%
13.	Sadao/2nd patient house	OP698063	*L. martiniquensis*	CSP45	*C. oxystoma*	*L. martiniquensis* 770605_Trang (KY982650), 99.61%
14.	Sadao/2nd patient house	OP698064	*L. martiniquensis*	CSP48	*C. oxystoma*	*L. martiniquensis* 770605_Trang (KY982650), 99.61%
15.	Sadao/2nd patient house	OP698065	*L. martiniquensis*	CSP59	*C. mahasarak-hamense*	*L. martiniquensis* SK4-1 (MK603826), 100%
16.	Rattaphum/livestock sheds	OP698066	*L. martiniquensis*	CHH10	*C. fordae*	*L. martiniquensis* 770605_Trang (KY982650), 100%
17.	Rattaphum/livestock sheds	OP698067	*L. martiniquensis*	CHH45	*C. fulvus*	*L. martiniquensis* 770605_Trang (KY982650), 100%

Table S3. The BLASTn result of *Crithidia SSU rRNA* sequences derived from field-caught. Culicoides biting midges in this study.

No.	Location	Accession no.	Parasite Identi-fication	Isolate	Host spe-cies	Top BLASTn Matchesand % Similarity
1.	Sadao/2nd patient house	OP698037	*Crithidia* sp.	CSP18	*C. peregrinus*	*C. thermophila/confusa/deanei*, 97.21%
2.	Sadao/2nd patient house	OP698038	*Crithidia* sp.	CSP20	*C. peregrinus*	*C. thermophila/confusa/deanei*, 97.42%
3.	Sadao/2nd patient house	OP698039	*Crithidia* sp.	CSP22	*C. peregrinus*	*C. thermophila/confusa/deanei*, 96.78%
4.	Sadao/2nd patient house	OP698040	*Crithidia* sp.	CSP25	*C. peregrinus*	*C. thermophila/confusa/deanei*, 96.99%
5.	Sadao/2nd patient house	OP698041	*Crithidia* sp.	CSP28	*C. peregrinus*	*C. thermophila/confusa/deanei*, 97.31%
6.	Sadao/2nd patient house	OP698042	*Crithidia* sp.	CSP30	*C. peregrinus*	*C. thermophila/confusa/deanei*, 96.46%
7.	Sadao/2nd patient house	OP698043	*Crithidia* sp.	CSP42	*C. oxystoma*	*C. thermophila/confusa/deanei*, 96.99%

8.	Sadao/2nd patient house	OP698044	*Crithidia* sp.	CSP50	*C. oxystoma*	*C. thermophila/confusa/deanei*, 96.24%
9.	Rattaphum/livestock sheds	OP698045	*Crithidia* sp.	CHH64	*C. fordae*	*C. thermophila/confusa/deanei*, 97.31%
10.	Rattaphum/livestock sheds	OP698046	*Crithidia* sp.	CHH65	*C. fordae*	*C. thermophila/confusa/deanei*, 96.89%
11.	Rattaphum/livestock sheds	OP698047	*Crithidia* sp.	CHH95	*C. orientalis*	*C. thermophila/confusa/deanei*, 96.89%
12.	Rattaphum/livestock sheds	OP698048	*Crithidia* sp.	CHH113	*C. elbeli*	*C. thermophila/confusa/deanei*, 97.42%

Accession nos of C. thermophila/confusa/deanei references were KY264937, JF717837, andEU079129, respectively.
